# Fibrotic Hypersensitivity Pneumonitis: Key Issues in Diagnosis and Management

**DOI:** 10.3390/jcm6060062

**Published:** 2017-06-15

**Authors:** Vasileios Kouranos, Joseph Jacob, Andrew Nicholson, Elisabetta Renzoni

**Affiliations:** 1 Interstitial Lung Disease Unit, Royal Brompton Hospital, National Heart and Lung Institute, Imperial College, Sydney Street, SW3 6NP London, UK; e.renzoni@imperial.ac.uk; 2 Department of Radiology, Royal Brompton Hospital, London, UK; joseph.jacob@nhs.net; 3 Department of Histopathology, Royal Brompton Hospital, and National Heart and Lung Institute, Imperial College, London, UK; A.Nicholson@rbht.nhs.uk

**Keywords:** fibrotic hypersensitivity pneumonitis, idiopathic pulmonary fibrosis, diagnosis, prognosis

## Abstract

The diagnosis of hypersensitivity pneumonitis (HP) relies on the clinical evaluation of a number of features, including a history of significant exposure to potentially causative antigens, physical examination, chest CT scan appearances, bronchoalveolar lavage lymphocytosis, and, in selected cases, histology. The presence of fibrosis is associated with higher morbidity and mortality. Differentiating fibrotic HP from the idiopathic interstitial pneumonias can be a challenge. Furthermore, even in the context of a clear diagnosis of fibrotic HP, the disease behaviour can parallel that of idiopathic pulmonary fibrosis in a subgroup, with inexorable progression despite treatment. We review the current knowledge on the diagnosis, management, and prognosis of HP with particular focus on the fibrotic phenotype.

## 1. Introduction

Hypersensitivity pneumonitis (HP), also known as extrinsic allergic alveolitis, is a clinical syndrome characterized by variable presentations (acute, subacute, and chronic/fibrotic). Frequently, an overlap of the different disease forms is observed in daily clinical practice. An exaggerated immune response to repeated inhalation of environmental antigens is the proposed pathogenetic mechanism [1]. As only a small proportion of individuals exposed to specific antigens develop the disease, and contributing factors of a predisposed genetic background are likely to be implicated. Critical immunopathological changes occur in the lung microenvironment. In fibrotic HP, ongoing immune activation and inflammation are believed to lead to the expansion/activation of the fibroblast population and the accumulation of extracellular matrix [2,3]. The reasons why only some individuals develop progressive fibrosis in HP are not known. Although our knowledge of the pathogenesis and the list of environmental antigens associated with HP has continued to increase, the clinical definition of the disease remains elusive. Two major attempts to reach an agreement on a definition by groups of international experts failed to provide a widely accepted and validated diagnostic approach [1,4].

In this context, symptoms, radiographic and histopathological findings in patients with fibrotic HP often overlap with those described in patients with idiopathic interstitial pneumonias (IIPs), as HP findings of bronchiolocentric granulomatous inflammation are often associated with a background pattern of usual interstitial pneumonia (UIP) or non-specific interstitial pneumonia (NSIP) [5,6]. Indeed, the updated diagnostic guidelines for idiopathic pulmonary fibrosis (IPF) recommend the exclusion of fibrotic HP before reaching an IPF diagnosis [7]. The challenges faced in diagnosing HP are highlighted by a recent multi-centre study showing the poor inter-multidisciplinary team agreement in diagnosing HP (weighted kappa = 0.29; interquartile range (IQR) 0.24–0.4), compared to a markedly better agreement between different multidisciplinary teams (MDTs) in diagnosing IPF (weighted kappa = 0.71; IQR 0.64–0.77) [8]. From a therapeutic management perspective, the distinction between IPF and an inflammation-driven disorder such as fibrotic HP is crucial, since immunosuppressive therapy is considered to be harmful in IPF [9], but may be appropriate in fibrotic HP. Conversely, anti-fibrotic regimens have been licensed for IPF, but not for fibrotic HP.

In HP, the presence of fibrosis histologically or on chest computer tomography (CT) is associated with a decreased survival. In view of this and of the diagnostic difficulties in separating fibrotic HP from IPF or other interstitial lung diseases (ILDs) in a proportion of patients, this review article focuses on fibrotic HP with particular attention to current knowledge on the diagnosis, management, and prognosis of this form of the disease. In addition, steps in the diagnostic approach that may be helpful in distinguishing fibrotic HP from IPF are discussed. For the purpose of this review we will use the term fibrotic HP rather than chronic HP, as there can be patients with a chronic subacute form without significant fibrosis visible on CT.

## 2. Diagnosis of Fibrotic HP

The diagnosis of fibrotic HP relies on the integration of: (a) non-specific symptoms, such as dyspnea and cough, as well as fatigue and malaise, that develop in a specific environment, (b) compatible chest CT scan features, (c) detection of serum antibodies against suspected antigens, (d) lymphocytosis on bronchoalveolar lavage (BAL), and/or (e) identification of a granulomatous bronchiolocentric interstitial pneumonitis on adequate lung biopsies. None of the above features is specific for the diagnosis of HP in isolation. Although internationally-agreed guidelines are lacking, a diagnosis of HP is based on variable combinations of the above features in the individual patient. We will detail each of the features that can lead to a diagnosis of fibrotic HP below.

### 2.1. Exposures

A careful and thorough exposure history is key in the diagnostic approach of the individual patient. Since the first description of farmer’s lung in the 60 s, the wide list of agents associated with the development of HP has continued to expand, including fungal, bacterial, protozoal, and animal proteins, as well as low-molecular-weight chemical compounds. However, the most common causes of HP are exposure to avian antigens, or to fungi/actinomyces in the home or in the working environment [1].

The classic association with avian antigen exposure occurs in pigeon breeders [10,11], or in individuals keeping parakeets/cockatiels as pets in their homes [12], although exposure can be more subtle, and cases of HP related to birds visiting areas adjacent to the home [13], or even in feather pillows, duvets or mattress covers have been described [14]. Avian antigens can remain in the house long after the removal of the birds themselves, something that needs to be considered when counselling the patient [12,13,15].

Visible mould and known water damage for a number of reasons (e.g., floods, leaks) in the home or the occupational environment have been associated with HP, as they are related to inhalation of specific microbiologic agents [16]. In addition, questions about exposures to greenhouses, compost, mushroom farming, and other food production methods where mould growth can occur should be included in the patient’s detailed history [17,18,19,20]. More recently, hot tub lung and metalworking fluid-related HP have been described [21,22,23]. Non-tuberculous mycobacteria have been identified in patients exposed to indoor hot tubs and outdoor pools [21], and metal working fluids can be contaminated by bacterial, mycobacterial, and fungal organisms [23]. Finally, specific chemicals used in industry, such as isocyanates and anhydrides, should also be considered as causal antigens. A more detailed list of HP antigens can be found in dedicated reviews [24]. An example of how an exposure can be missed if not specifically considered, is the case of a patient attending the Royal Brompton ILD clinic with clinical and radiological features consistent with fibrotic HP. The patient had been managed for fibrotic HP for a few years, but it was only when he mentioned that his symptoms improved when on holiday that a work exposure was queried. A bronchoalveolar lavage performed after he had returned to work post-holidays revealed marked lymphocytosis of 54%, suggestive of ongoing exposure and inflammation, as well as mild neutrophilia (5.6%). A detailed occupational history revealed exposure to metalworking fluids in an aircraft factory, confirmed by an inspection of his work environment, and gradual symptomatic and functional improvement was noted since removing the exposure and introducing immunosuppressive treatment.

The time interval between the onset of the exposure and the onset of disease is variable and may range from months to decades [25]. Patients in whom the offending antigen is identified have a better prognosis [15]. However, disease progression even after termination of the antigen exposure often occurs. Furthermore, finding the inciting antigen is extremely difficult in fibrotic HP. In a series of 85 consecutive cases, no antigen was identified in 25% [16], while in another series of 142 cases; a responsible antigen was not detected in 53% of the patients [15]. These studies illustrate the challenges faced and the importance of determining the relevant exposure, not just for the management of fibrotic HP, but also in the evaluation of fibrotic lung diseases overall. A thorough exposure history looking for exposures associated with HP, including attention to seemingly minor exposures is, thus, an essential part of the evaluation of patients presenting with diffuse parenchymal lung disease.

### 2.2. High Resolution CT Findings

Chest CT evaluation forms a central component of the diagnostic work-up of a patient with fibrotic lung disease. In subacute HP, ground-glass opacities that are considered to represent lymphocytic alveolar inflammation and organizing pneumonia can be seen alongside poorly-defined centrilobular nodules, which are thought to reflect cellular bronchiolitis [26,27,28].

**Figure 1:**
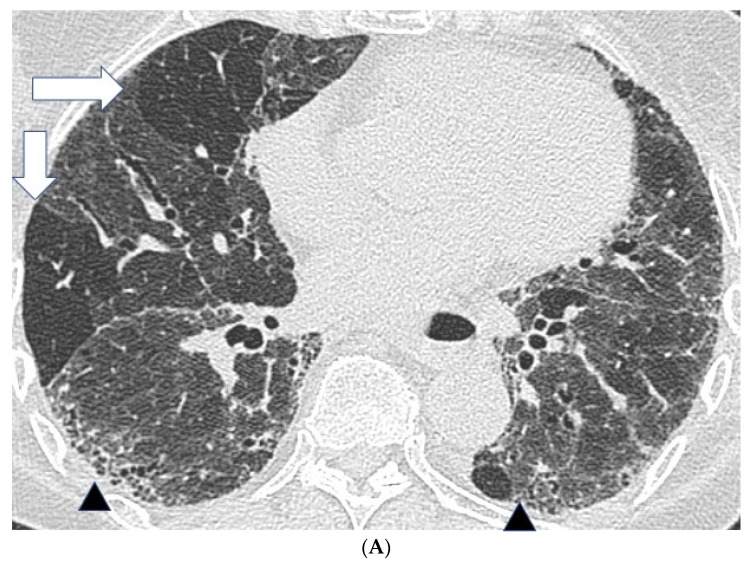

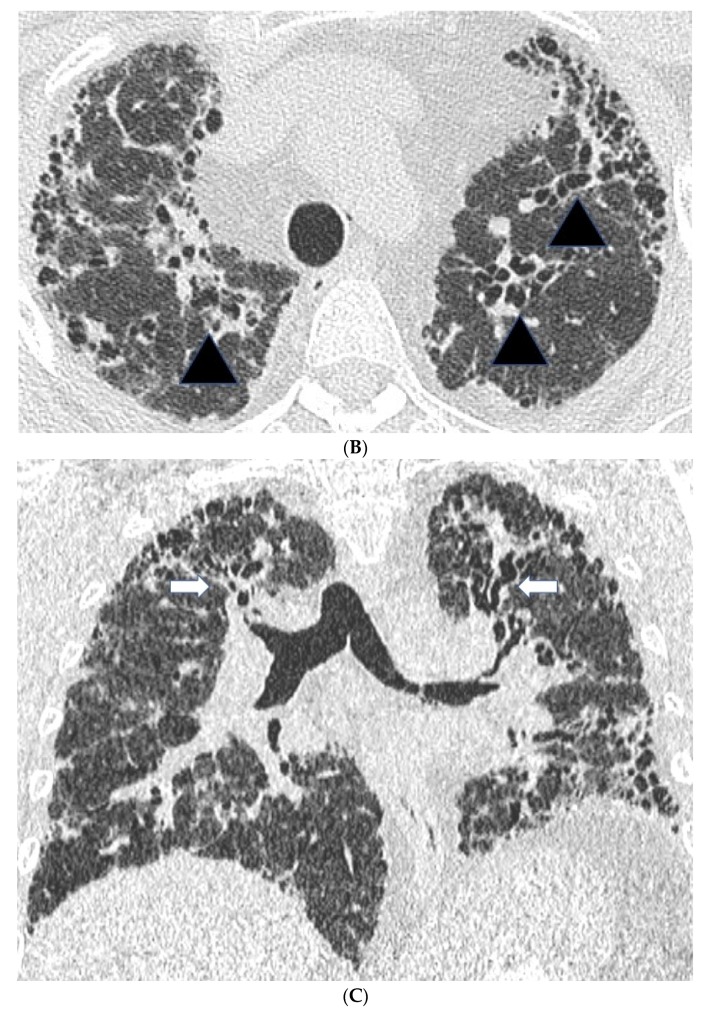

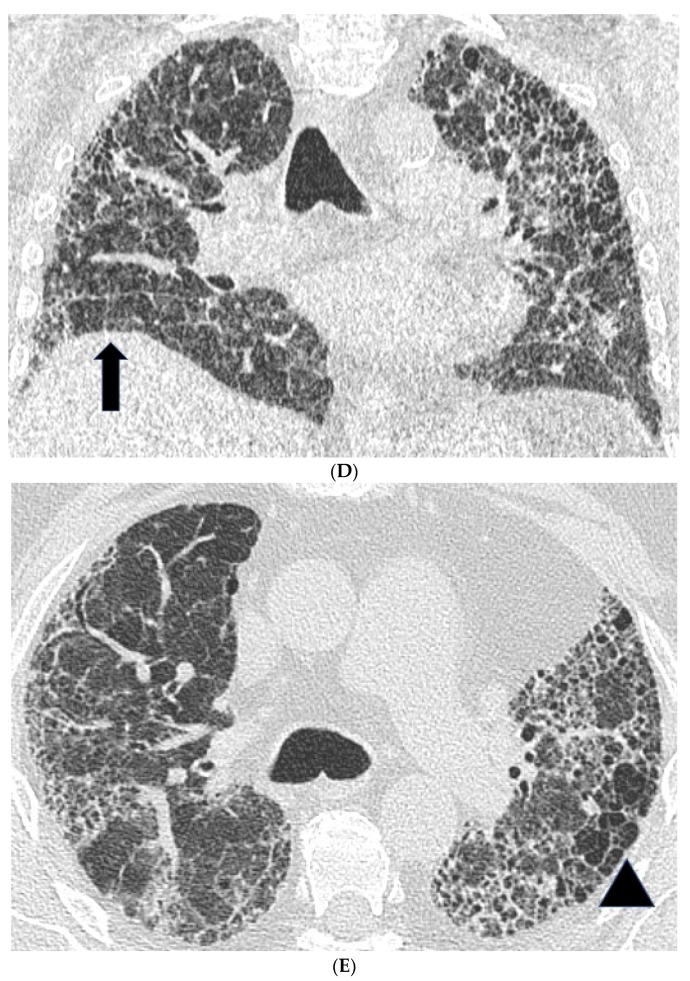
Features suggestive of chronic hypersensitivity pneumonitis in different patients. (**A**) Spared pulmonary lobules are visible within non-fibrotic lung bilaterally (arrows); background fibrosis is evidenced by peripheral reticulation and traction bronchiectasis (black arrowheads). (**B**,**C**) A predilection towards bronchocentricity of the fibrosis in the upper lobes is evident on axial (arrowheads) and coronal (arrows) images. (**D**) An upper and midzone predominance to the fibrosis, characterised by reticulation and traction bronchiectasis, is seen on a coronal CT. Incidentally, volume loss in the right lung is noticeable by slight tenting and elevation of the right hemidiaphragm (arrow). (**E**) A UIP pattern with honeycomb cysts is visible in the left midzone of the lung (arrowhead) in a 74-year-old male ex-smoker. A surgical biopsy performed a few years before this CT had demonstrated findings compatible with fibrotic hypersensitivity pneumonitis.

In fibrotic HP, CT patterns including reticulation, traction bronchiectasis and volume loss, with or without evidence of honeycombing, are all seen (Figure 1) [26,28]. Patients often demonstrate an upper lobe predominant distribution of fibrosis, but diffuse and lower lobe predominant changes have also been described. Furthermore, a faint bronchocentricity to the fibrosis can be observed at the lung apices. Similarly, reticulation in fibrotic HP is considered to have a predominantly subpleural or peri-bronchovascular distribution.

In a study of 92 patients with fibrotic HP, ground glass opacification and reticulation were the most prevalent patterns of interstitial disease [27]. Interestingly, microcystic and macrocystic honeycombing were described in 32% and 13% patients, respectively. The prevalence of traction bronchiectasis was greater within regions of ground glass opacification and reticular patterns than within areas of honeycombing.

Mosaic attenuation with patchy areas of air trapping in a lobular distribution, more obvious on expiratory scans, is another important CT feature in HP. Lobular air trapping represents indirect evidence of small airways obstruction. (Figure 1A) However, mosaic attenuation can also be observed in other fibrotic lung diseases, including fibrotic sarcoidosis and connective tissue disease associated interstitial lung disease, also related to small airways involvement. As a result, lobular air trapping, which is often considered characteristic, may be a relatively non-specific finding. Interlobular septal thickening can be particularly profuse in fibrotic HP, although this is also a feature of fibrotic sarcoidosis. Consolidation is rare in HP and should raise the suspicion of alternative diagnoses such as fibrosing organizing pneumonia or sarcoidosis. Finally, cysts have been reported in 13% of patients with subacute HP but can also be seen in fibrotic HP [29]. The cysts are suspected to be a result of bronchiolar obstruction secondary to peribronchiolar lymphocytic inflammation, similar to that seen in lymphoid interstitial pneumonia.

Several of the chest CT features mentioned above, suggestive of a fibrotic HP diagnosis, have been used to classify patients as having features inconsistent with a CT UIP pattern in the latest consensus IPF diagnostic guidelines [7]. Specifically, the presence of extensive lobular air trapping, centrilobular nodules, and the lack of a lower zone predominance to the fibrosis can be helpful in excluding IPF as a working diagnosis (Table 1). Conversely, changes that are diffuse, but predominantly lower lobe in distribution, and where areas of mosaic attenuation are not extensive (unilateral or involving less than three lobes), make the diagnostic separation of fibrotic HP and IPF challenging. In a study of 66 patients with fibrotic interstitial lung disease, a clear distinction between fibrotic HP and other fibrotic idiopathic interstitial pneumonias on thin section CT was possible in only 53% of patients [30]. The diagnostic difficulties were further highlighted by an international multicenter evaluation of 70 patients discussed in a series of parallel multidisciplinary team meetings [8]. The inter-multidisciplinary meeting discussion agreement among specialists on the diagnosis of hypersensitivity pneumonitis was the lowest among ILD patterns (weighted kappa = 0.29 (0.24–0.40)), emphasizing the lack of confidence even among ILD experts in the diagnosis of this entity.

Chest CT imaging in fibrotic HP can provide useful information regarding patient prognosis. Several studies have demonstrated that the presence and extent of fibrosis is predictive of mortality in HP [27,31,32]. In a mixed study group of 69 patients with subacute and chronic HP, severe impairment of pulmonary function and CT features of fibrosis were associated with a poor prognosis [31]. A study of 92 patients with fibrotic HP concluded that an increased extent of fibrosis on CT conferred a worse prognosis. The severity of traction bronchiectasis was found to be the strongest predictor of mortality, and remained so after adjustment for disease severity. Traction bronchiectasis severity was also shown to be superior to lung function tests in prognostic determination [27]. New quantitative imaging techniques using automated computer-based CT (CALIPER) analysis have recently shown that the extent of reticulation on CT is an independent predictor of mortality in fibrotic HP [32].

### 2.3. Serum Antibodies against Suspected Antigens

Positive precipitating antibodies to the offending antigen were considered in the clinical diagnosis of HP by the HP Study Group in 2003 [1]. Positive precipitins were considered supportive of the diagnosis in the appropriate clinical setting, since the presence of specific IgG antibodies to the inducing antigen in isolation is evidence of sensitization but not necessarily of disease. In addition, the significance of positive antibodies may differ according to exposure type. For example, healthy farmers have been found to have precipitating antibodies to exposure antigens in 30% to 60% of cases [33]. On the other hand, only 3% of healthy exposed budgerigar fanciers produce precipitating antibodies [34]. In a significant proportion of patients, no serum precipitins are identified to support the diagnosis of HP. Despite these limitations, the precipitin assay is a useful additional laboratory test in the diagnostic assessment of HP, in particular to suggest a potential exposure that may have not been recognized. Having said this, the newer ELISA-based assays are more sensitive, but less specific, than the traditional precipitating antibody assays, and the finding of a positive result must always be interpreted in the context of the individual patient’s exposures [24].

A natural challenge at the workplace or home, or a “provoked” inhalation challenge under standardized conditions after a period of avoidance, has been used in a few studies to support the diagnosis of HP and identify the specific antigen [35,36,37,38]. The lack of standardized antigens and challenge techniques has restricted the widespread use of such techniques.

### 2.4. Bronchoalveolar Lavage (BAL)

BAL is overall a well-tolerated interventional diagnostic procedure. It is widely used as a highly-sensitive diagnostic tool to detect alveolar inflammation in patients with interstitial lung disease. BAL has established diagnostic value in the diagnosis of HP, sarcoidosis, infections, and malignancy, which remain in the differential diagnosis of patients with idiopathic interstitial pneumonias. HP is characterized by an increase in the total cell count with elevation in the percentage of lymphocytes. The exact level of lymphocytes that differentiates HP from other disorders is not known, but in published series an increase in lymphocytes >50% is highly indicative [5,39]. A CD4/CD8 ratio <1 has also been considered suggestive of the diagnosis [40]. However, CD4/CD8 ratio measurements are no longer recommended, as the ratio may vary significantly according to exposure and disease stage. In addition, a normal or elevated ratio does not exclude HP [41,42]. With regards to the cytology cell differential count, it should be noted that acute episodes of exposure to relevant antigens may be associated with an increase in the number of neutrophils, reverting back to the predominantly lymphocytic profile after a few days [42]. On patient follow-up, the finding of a persistent BAL lymphocytosis may indicate that complete antigen exposure elimination has not been achieved.

Although the 2002 ATS/ERS international consensus classification of idiopathic interstitial pneumonias (IIPs) did not consider BAL analysis essential in the diagnostic workup of IPF [43], this remains a key element in the diagnosis/exclusion of fibrotic HP. However, the alveolar inflammation noted in patients with HP is less prominent when the disease is fibrotic [5,39]. Ohtani et al. reported a mean lymphocyte count of 19% when a UIP pattern on CT was evident in patients with fibrotic HP [39], while another series reported a mean BAL lymphocytosis of 36% in patients with a UIP pattern on CT, compared to 65% in those with a typical subacute HP pattern on CT [5]. There is no consensus on the exact percentage of BAL lymphocytes threshold useful to differentiate fibrotic HP from IPF. In a study of 74 patients with suspected IPF on chest CT, a BAL lymphocytosis cut-off threshold of 30% managed to differentiate 6/74 (8%) of patients from IPF, results that were later on confirmed on surgical biopsies and/or disease behaviour [44]. The mean lymphocyte count in BAL of patients with IPF is 11% and the vast majority of IPF patients have less than 20% lymphocytes [45]. These findings would suggest that fibrotic HP is highly likely in a patient with evidence of fibrotic lung disease and an elevated BAL lymphocyte count even if the HRCT is suggestive of a UIP pattern, although further studies are needed. Surgical lung biopsies may be efficacious in this setting but other important factors will also contribute to the final diagnosis. The study by Morell et al. reporting that among 46 patients with a diagnosis of IPF, a subsequent diagnosis of fibrotic HP was made in 20 patients, would imply that BAL should be performed routinely in all patients with fibrotic interstitial lung disease [46].

### 2.5. Histopathology

Although in a large proportion of cases, the diagnosis of HP can be reached through the criteria outlined above, a surgical lung biopsy is required in cases with low to moderate pretest likelihood of the disease diagnosis. This occurs mostly in patients with overlap of subacute/fibrotic HP findings on CT.

**Figure 2:**
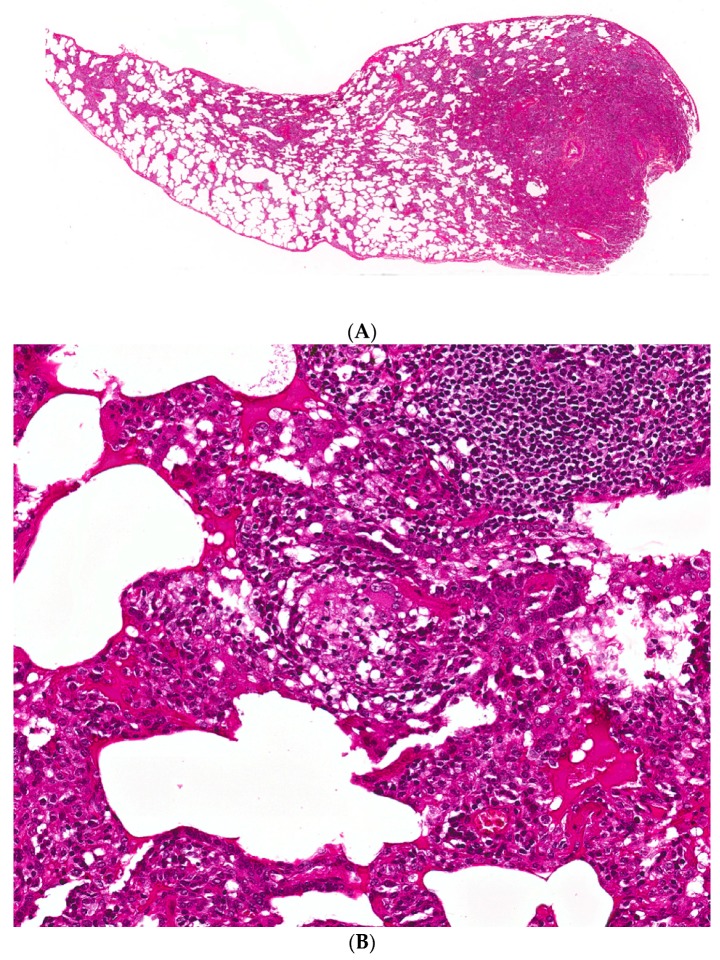
(**A**) A case of hypersensitivity pneumonitis shows peribronchiolar chronic inflammation of varying intensity around bronchovascular bundles. (**B**) A single, small, poorly-formed granuloma is found within the interstitium

The primary histopathological features considered characteristic of subacute HP include the triad of cellular bronchiolitis, lymphoplasmacytic interstitial infiltrates, and non-necrotizing granulomas (Figure 2) [47]. The inflammation is composed mainly of lymphocytes in a bronchiolocentric distribution. Proliferative bronchiolitis obliterans has been described in patients with farmer’s lung, while constrictive bronchiolitis has been reported in pigeon breeder’s disease [48,49]. The granulomas are small in size and not as well-formed as those seen in sarcoidosis. Multinucleated giant cells may be present as constituents of granulomas and/or also be present singly, not infrequently containing cholesterol clefts. It should be noted that granulomatous features may be absent in almost one third of surgical lung biopsies from patients with fibrotic HP [50]. Most granulomas are identifiable on routine staining, although cathepsin K, a protease expressed in activated macrophages, may be of additional value. However, it is not specific for HP granulomas [51]. Finally, occasional areas of organizing pneumonia with Masson bodies are often seen in subacute damage, while peribronchial lymphoid hyperplasia is frequently evident [50].

**Figure 3:**
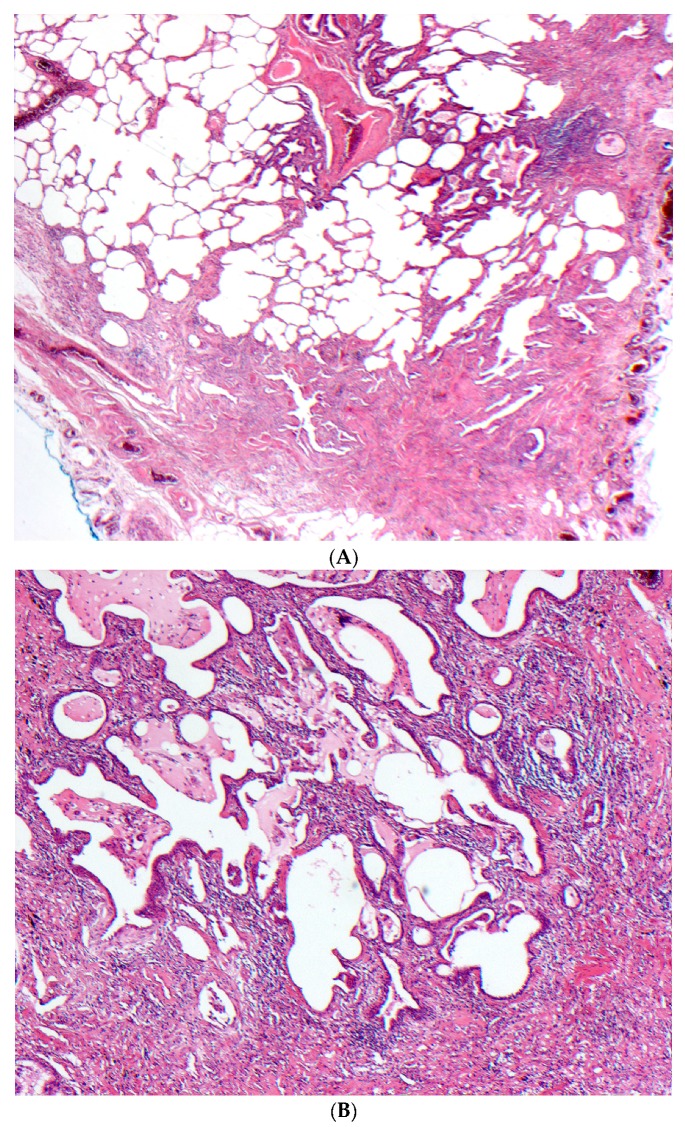

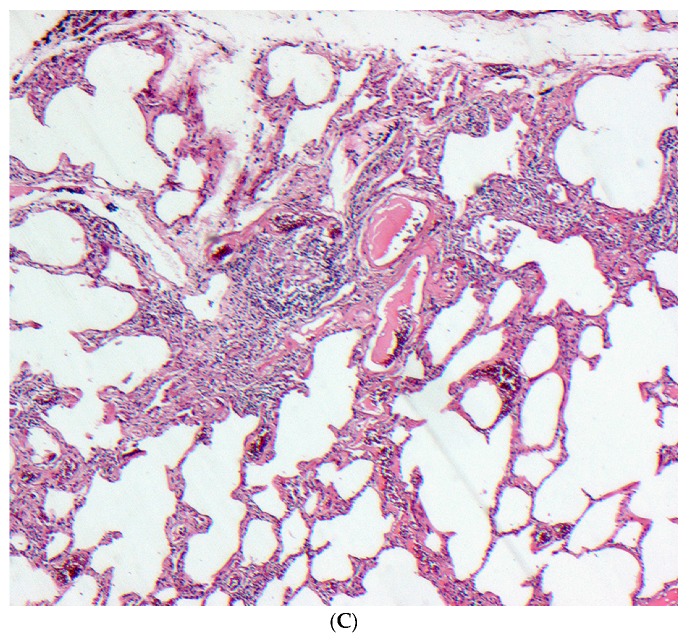
(**A**–**C**) A case of chronic hypersensitivity pneumonitis shows (**A**) areas of patchy subpleural dense fibrosis and (**B**) focal honeycomb change, typical of a pattern of usual interstitial pneumonia. (**C**) Rare peribronchiolar granulomas are also seen.

The histopathological patterns observed in fibrotic HP are characterized by variable degrees of fibrotic changes and are considered similar to those of NSIP or UIP in up to 88% of cases (Figure 3) [52,53,54]. Detecting fibrotic changes on biopsy is crucial as the presence of fibrosis is an independent predictor of morbidity and mortality in HP. Histopathologic patterns of fibrotic NSIP and UIP pattern in 119 patients were significantly correlated with a worse transplant-free survival time [55], while the presence of fibroblast foci on biopsies, regardless of the underlying histopathologic pattern, was found to be an independent predictor of survival in HP patients in two studies [55,56]. Evidence of organizing pneumonia and/or cellular NSIP pattern in surgical lung biopsies of patients with chronic bird fancier’s disease was most often noted in acute episodes of the disease and resulted in a more favourable outcome compared to patients with fibrotic NSIP and/or UIP pattern [39]. In one study, the median survival of fibrotic HP varied between 5–9 years depending on the identification of the inciting antigen [15], a survival that is considerably lower than the one reported for non-fibrotic cases.

#### 2.5.1. Distinguishing Fibrotic HP Associated with a UIP/Fibrotic NSIP Pattern from UIP/IPF

In patients with a CT consistent with fibrotic NSIP/UIP pattern, in whom HP is suspected, a biopsy can be helpful in differentiating fibrotic HP from IPF. The bronchiolocentric distribution of the fibrotic changes and the presence of signs of granulomatous inflammation, including giant cells, or granulomas, are supportive of the diagnosis of fibrotic HP. However, there are limitations. None of the features above are specific for HP, as each in isolation can also be found in IPF. On the other hand, the granulomatous inflammation becomes less prominent as the fibrotic disease progresses. In one study, 8% of cases were indistinguishable from UIP/IPF on histopathological analysis [57]. It remains unclear whether fibrotic HP with a UIP pattern on biopsy behaves differently from idiopathic UIP/IPF [58]. In a study of 16 patients with fibrotic HP and a UIP pattern on biopsy, there were no lymphocytosis on BAL and the distribution of fibrosis on chest CT scans was less predominant in 50% of patients while no zonal predominance was identified in 37.5% of patients [58]. The extent of fibrosis was significantly correlated with an increased risk for mortality (hazard ratio, 2.36; 95% CI, 1.02–5.48; *p* = 0.04) and there was a trend for an inverse association with acute exacerbation-free interval.

#### 2.5.2. Cryobiopsy

In view of the risks of a surgical biopsy, alternative ways of obtaining lung tissue for histology have been developed. Conventional transbronchial biopsies are associated with a low diagnostic yield, related to the small sample size, coupled with the patchy nature of interstitial involvement. Increasingly, transbronchial cryobiopsies are being used in a number of ILD centres, as the technique allows for larger sample size, fewer crush artefacts and, therefore, higher diagnostic yields compared to the conventional transbronchial biopsies. A significantly lower mortality rate in expert centres compared to a surgical lung biopsy (<0.5% vs. 2–4%) was recently reported [59]. In another recent study comparing the diagnostic accuracy and safety profile of transbronchial cryobiopsy and video-assisted thoracoscopic (VATS) lung biopsy, Iftikhar et al. showed that the pooled diagnostic yield was comparatively lower than VATS lung biopsy, although not markedly so (83.7% vs. 92.7%), while the incidence of significant bleeding and pneumothorax post the transbronchial biopsy was considered acceptable (4.9% and 9.5%, respectively) [60]. However, the specific diagnostic yield of a cryobiopsy in relation to distinguishing UIP associated with HP versus UIP/IPF remains to be established.

## 3. Management of Fibrotic HP

Management decisions in HP can involve modification of both the environment (antigen avoidance) and the host immune response. Avoidance of antigen exposure is often effective in cases of acute and sub-acute HP, but is usually insufficient in isolation in fibrotic HP. Fibrotic HP is widely considered to be inflammation driven and, thus, immunosuppression tends to be used, especially in cases with disease progression. The underlying assumption is that the disease regression seen with corticosteroids in cases of acute and sub-acute HP can be extrapolated to the prevention or reduction of progression in chronic HP. However, no controlled data exist to confirm this view and there is no consensus on an optimal regimen, including the level and duration of corticosteroid and/or immunosuppressive therapy. Furthermore, given the frequent difficulty in distinguishing between chronic HP and IPF, there is a concern that high dose corticosteroids/immunosuppression may have deleterious effects in some patients with a provisional diagnosis of fibrotic HP who do, in reality, have IPF [9].

In view of the significant side effects of long term high dose corticosteroid treatment, mycophenolate mofetil (MMF) and azathioprine (AZA) are frequently used as steroid-sparing agents. Treatment with MMF or AZA was associated with a statistically significant improvement in gas transfer values after one year of treatment in a recent study comparing the two regimens in patients with fibrotic HP [61]. More intensive immunosuppression has been used in cases of rapidly progressive non-IPF interstitial lung disease. One case with admixed subacute/fibrotic HP experienced a dramatic improvement in symptoms and lung function post Rituximab, despite failure to respond to intravenous cyclophosphamide [62]. In the study by Keir et al., six patients with fibrotic HP whose lung function tests had declined despite conventional immunosuppressive therapy were treated with rituximab [63]. Following rituximab, pulmonary function tests stabilized or improved in three patients out of six and continued to deteriorate in the other three patients, all of whom died within four months of treatment.

Prospective randomized trials are needed to validate the effectiveness of the available immunosuppressive regimens in patients with fibrotic HP. On the other hand, it is clear that some patients with fibrotic HP have a longitudinal behaviour akin to IPF, with relentless ongoing progression and early mortality despite immunosuppression. At the moment, there is no means of determining which patients with fibrotic HP will follow an IPF-like course. High throughput studies investigating genomic, epigenomic, and proteomic biomarkers in fibrotic HP and other fibrotic ILDs are needed to improve diagnostic and prognostic accuracy, in combination with current CT and histologic criteria. The use of anti-fibrotic drugs such as pirfenidone and nintedanib, proven to slow down functional decline in IPF, has been suggested as an option for patients with progressive fibrotic HP and no documented response to corticosteroids and/or immunosuppressants [64]. A number of randomized placebo-controlled trials are currently recruiting in non-IPF progressive fibrosis patient groups, including a trial evaluating the effects of pirfenidone as an add-on drug to background immunosuppressive treatment in patients with fibrotic HP (clinical trial gov. number: NCT02496182) [65], and a study (clinical trial gov. number: NCT02999178) is evaluating the effects of nintedanib compared to placebo in a wider group of patients with non-IPF progressive fibrotic lung disease, including fibrotic HP [66].

## 4. Conclusions

The development and the clinical patterns of HP are influenced by a number of factors, including: (a) the nature/amount of inhaled antigens, (b) the intensity and frequency of exposure, and (c) the host immune/fibrotic response, in turn likely influenced by a predisposing genetic background. An accurate diagnosis of fibrotic HP is essential, as fibrotic HP requires a distinct management from IPF and is largely linked with a better disease outcome. However, a proportion of patients with fibrotic HP have an IPF-like disease progression and survival, and may benefit from anti-fibrotic drugs, to be tested in appropriately powered clinical trials. Further knowledge is needed on the molecular pathways underlying progressive fibrosis in fibrotic HP and the different phenotypes of the disease, so as to allow: (a) early detection and intervention to prevent disease progression to irreversible fibrosis; (b) improved targeting of available immunosuppressive and/or anti-fibrotic agents to the appropriate patient subgroups; and (c) identification of novel targets for more effective treatment.

## Figures and Tables

**Table 1:** Similarities and differences in clinical, radiological and histopathological features between fibrotic HP and idiopathic pulmonary fibrosis (IPF).

Features	Fibrotic HP	IPF
*Demographics*		
Sex	No difference	More frequent in men
Smoking	Protective	Risk factor
Age	No predilection	More frequent > 55 years
*Clinical symptoms/history*		
Clubbing	Often	Often
Squeaks	Typical	Absent
Bibasal crackles	Frequent	Frequent
Systemic disease features (fever, joint pains, fatigue)	Often	Absent
Exposure to antigens	Frequent	Rare
Positive precipitins	Frequent	Rare
*Imaging (CT)*		
Distribution	Upper lobe predominance	Peripheral, predominantly basal
Mosaic attenuation	Frequent	Absent/Limited
Nodules	Frequent	Absent
Interlobular septal thickening	Often	Absent/Limited
Honeycombing	Often	Frequent (typical for UIP pattern)
Bronchocentricity	Frequent	Absent
Discrete cysts	Often	Absent
Consolidation	Rare	Absent
*Bronchoalveolar lavage (BAL)*	Lymphocytosis > 25–30%	Lymphocytosis < 20%
*Histological*		
Fibroblast foci	Often	Frequent
Granulomas/giant cells/Schaumann bodies	Frequent	Rare
Organizing pneumonia	Rare	Rare
Honeycombing	Often	Frequent
Paraseptal subpleural distribution	Often	Frequent
Bronchocentricity	Frequent	Absent
